# Ultrasound-Guided Mini Fluid Challenge via Velocity–Time Integral to Assess Fluid Responsiveness in Upper Gastrointestinal Bleeding: A Prospective Quasi-Experimental Feasibility Study

**DOI:** 10.3390/life16081236

**Published:** 2026-07-27

**Authors:** Ilker Çermikli, Tufan Alatli, Salih Kocaoglu

**Affiliations:** 1 Department of Emergency Medicine, Sile State Hospital, Istanbul 34980, Türkiye; ilkercermikli@gmail.com; 2 Department of Emergency Medicine, Medicine Faculty, Balikesir University, Balikesir 10145, Türkiye; salihkocaoglu1986@gmail.com

**Keywords:** gastrointestinal bleeding, point-of-care ultrasound, mini fluid challenge, velocity–time integral, fluid responsiveness, Haemodynamics

## Abstract

**Background:** Acute upper gastrointestinal bleeding (UGIB) remains a leading cause of emergency department (ED) admission and carries a mortality of 5–10%. Static hemodynamic parameters perform poorly as predictors of fluid responsiveness, exposing patients either to under-resuscitation or to the harms of fluid overload. The mini fluid challenge (MFC), in which a 100 mL bolus is used to elicit a measurable change in the left ventricular outflow tract velocity–time integral (ΔVTI), is a validated dynamic test in intensive care but has not been evaluated in undifferentiated ED haemorrhagic shock. We aimed to determine whether ΔVTI-guided resuscitation is feasible at the bedside in UGIB and to explore how ΔVTI relates to in-hospital mortality. **Methods:** Prospective, single-centre, randomised quasi-experimental comparative study conducted between February 2024 and February 2025. Adults presenting with UGIB were allocated by treatment period, and credentialed emergency physicians to an ultrasound-guided protocol group (UGPG) or a standard care protocol group (SCPG). ΔVTI was measured in both groups but was disclosed to the treating team only in UGPG; in SCPG, it was recorded offline and analysed post hoc. The primary outcome was the feasibility of ΔVTI acquisition; the association of ΔVTI with in-hospital mortality was a secondary, exploratory outcome. **Results:** Eighty-five patients were enrolled (UGPG *n* = 47; SCPG *n* = 38). ΔVTI acquisition was completed within the intended time window in all enrolled patients, establishing bedside feasibility. Across the whole cohort, ΔVTI discriminated non-survivors poorly (AUC 0.55, 95% CI 0.44–0.66), and its performance differed markedly by group: AUC 0.83 (95% CI 0.67–0.93) in SCPG versus 0.69 (95% CI 0.54–0.82) in UGPG. In SCPG, non-survivors had substantially higher ΔVTI than survivors (58.6 ± 30.5% vs. 28.4 ± 30.9%; *p* = 0.030), a pattern consistent with persistent, uncorrected fluid responsiveness. In UGPG, the association ran in the opposite direction, mortality being concentrated among ΔVTI < 10% non-responders (*p* = 0.020). **Conclusions:** ΔVTI-guided resuscitation is feasible in the ED management of UGIB. The direction of the ΔVTI–mortality association differed according to whether ΔVTI was visible to clinicians, generating the testable hypothesis that unrecognised fluid responsiveness contributes to death in standard care. These findings are hypothesis-generating only: the small sample and the low number of events preclude any causal inference and require confirmation in an adequately powered, randomised, etiology-stratified trial.

## 1. Introduction

Acute upper gastrointestinal bleeding (UGIB) is a critical condition with a mortality rate ranging from 5% to 10% despite advances in endoscopic and pharmacological therapy [1,2,3]. The cornerstone of early management is hemodynamic stabilisation; however, fluid resuscitation in UGIB presents a unique “Goldilocks” problem. Aggressive fluid administration may precipitate dilutional coagulopathy, dislodge a forming clot (“pop the clot”) and raise portal pressure in variceal bleeding, whereas under-resuscitation leads to tissue hypoperfusion, organ failure, and increased mortality [4].

Traditional static parameters (blood pressure, heart rate, and central venous pressure) are poor discriminators of fluid responsiveness, because they describe the current circulatory state rather than the reserve of the ventricle to convert additional preload into stroke volume. Dynamic parameters address this directly by observing the hemodynamic response to a defined preload challenge, and thereby locate the patient on the ascending or the plateau portion of the Frank–Starling curve.

The mini fluid challenge and why ΔVTI; Stroke volume is the product of the left ventricular outflow tract (LVOT) cross-sectional area and the velocity–time integral (VTI) of blood traversing that tract. Since LVOT diameter is fixed over the short interval of a bolus, the change in VTI is a valid surrogate for the change in stroke volume and can be obtained non-invasively by pulsed-wave Doppler from an apical five-chamber view without the geometric assumptions required for absolute stroke-volume calculation. In the mini fluid challenge (MFC), a deliberately small bolus (100 mL of crystalloid infused over one minute) is administered, and VTI is re-measured one minute later. A rise of approximately 10% or more identifies a preload-dependent, “fluid-responsive” ventricle [5,6]. The small volume is the essential design feature: it is sufficient to displace a responsive ventricle along the steep portion of the Frank–Starling relationship, yet small enough that a non-responder incurs no meaningful fluid burden, a consideration of particular weight in UGIB, where the cost of unnecessary volume is not merely pulmonary but haemostatic.

Alternative dynamic tests are poorly suited to this specific population. Pulse-pressure and stroke-volume variation require controlled mechanical ventilation with adequate tidal volumes and sinus rhythm; conditions rarely met in a spontaneously breathing ED patient. The passive leg raise, although reversible, requires repositioning a patient who may be actively vomiting blood and at risk of aspiration. Inferior vena cava collapsibility is confounded by intra-abdominal pressure and by the marked respiratory effort common in haemorrhagic shock [7]. The MFC, by contrast, requires only a peripheral line, one minute of infusion, and two Doppler tracings. In intensive care and perioperative populations, the meta-analysis of Messina et al. reported pooled sensitivity and specificity in the range of 0.82–0.91 for the MFC [5], although its performance in undifferentiated ED haemorrhagic shock has never been prospectively evaluated.

Two gaps, therefore, motivated this study. First, whether the MFC can actually be executed within the operational constraints of a busy ED (competing resuscitation tasks, an unprepared patient, no dedicated sonographer) is unknown and is a question of feasibility rather than efficacy. Second, whether ΔVTI carries prognostic information in UGIB, and whether that information behaves differently when it is available to the treating clinician, is unexplored. This study was accordingly designed as a feasibility and hypothesis-generating investigation.

## 2. Methods

### 2.1. Study Design and Setting

**Figure 1:**
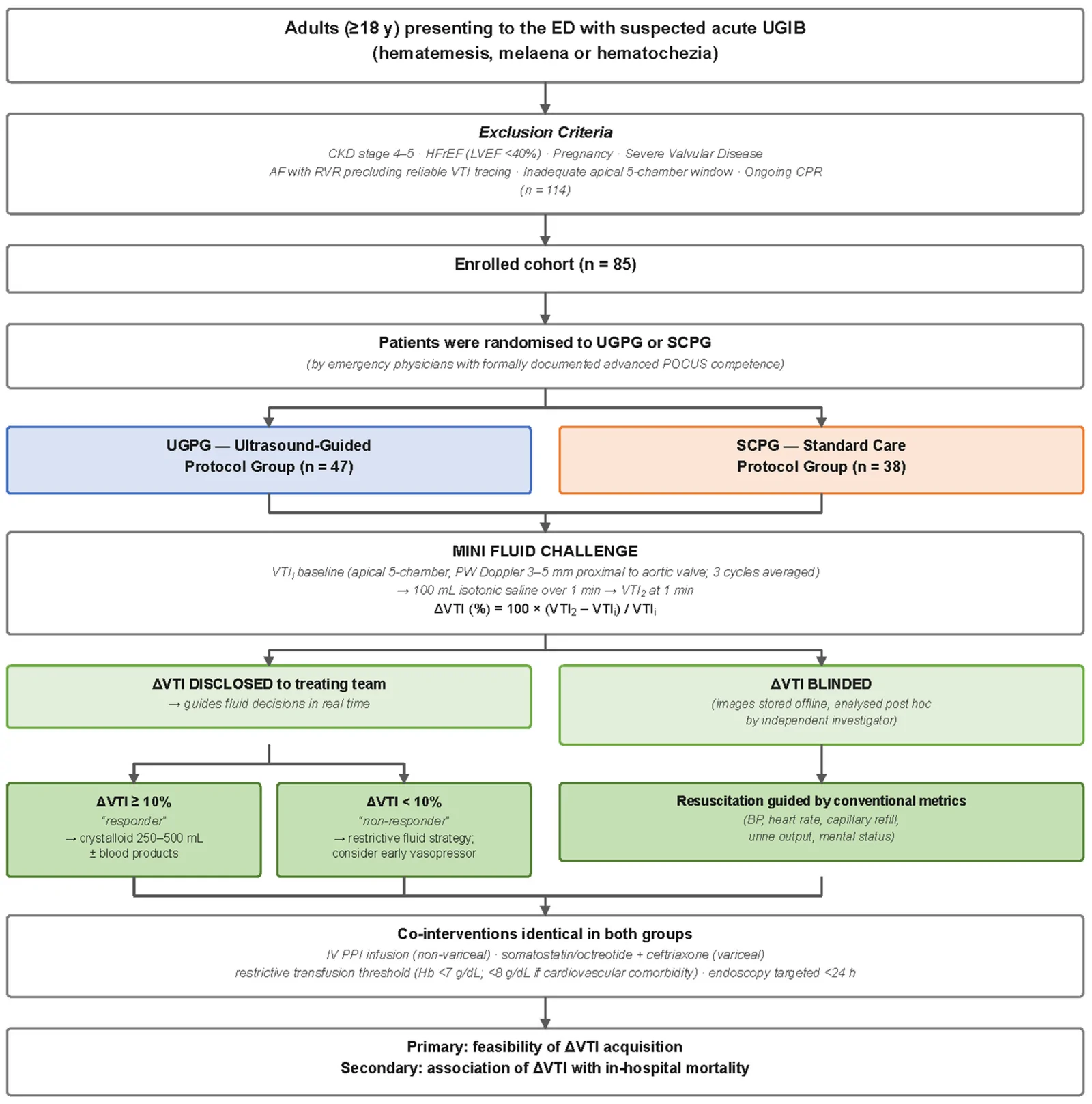
Schematic representation of the study protocol.

This prospective, single-centre, randomised quasi-experimental comparative study was conducted at the Emergency Department of Balıkesir University Faculty of Medicine, a tertiary care centre in Türkiye, from February 2024 to February 2025. The study protocol was approved by the Clinical Research Ethics Committee (Approval No: 2024/08) and adhered to the Declaration of Helsinki. Written informed consent was obtained from all participants or their legal surrogates. The complete study protocol is presented schematically in Figure 1.

### 2.2. Population

Inclusion criteria: patients aged ≥18 years presenting with clinical signs of UGIB (hematemesis, melena or haematochezia).

Exclusion criteria: chronic kidney disease (stage 4–5); heart failure with reduced ejection fraction (LVEF < 40%); pregnancy; known severe valvular disease; atrial fibrillation with rapid ventricular response precluding a reliable VTI tracing; inadequate apical five-chamber acoustic window; and patients requiring active cardiopulmonary resuscitation at presentation.

### 2.3. Group Allocation and Its Limitations

Patients were randomised to UGPG or SCPG by emergency physicians with formally documented advanced POCUS competence. ΔVTI was measured in all groups, but was strictly blinded to the treatment team in the SCPG group (images were stored off-line and analysed post hoc by an independent investigator).

This mechanism should be understood as a fundamental structural limitation, not a technical reservation. Therefore, the two groups are not directly comparable, and any observed difference in outcomes between the groups cannot be attributed to the intervention. For this reason, intergroup outcome comparisons are reported for the sake of completeness only and are expressly excluded from the interpretation of the study; all analyses of clinical interest are within-group.

### 2.4. Study Protocol and Groups

Ultrasound-Guided Protocol Group (UGPG): resuscitation was guided by ΔVTI measurements according to the algorithm detailed below and in Figure 1.

Standard Care Protocol Group (SCPG): resuscitation was guided by conventional metrics (blood pressure, heart rate, capillary refill, urine output, and mental status). ΔVTI measurements were performed in SCPG for blinded data analysis only and were never disclosed to the treating team.

Co-interventions were identical in both groups and followed institutional UGIB protocols aligned with international guidelines [4,8]: intravenous proton-pump inhibitor infusion in suspected non-variceal bleeding; somatostatin/octreotide and prophylactic ceftriaxone in suspected variceal bleeding in cirrhotic patients; and a restrictive transfusion threshold (haemoglobin <7 g/dL, or <8 g/dL in patients with cardiovascular comorbidity) per Villanueva et al. [9]. Endoscopy was targeted within 24 h of admission.

### 2.5. Ultrasound Measurement and Mini Fluid Challenge

**Figure 2:**
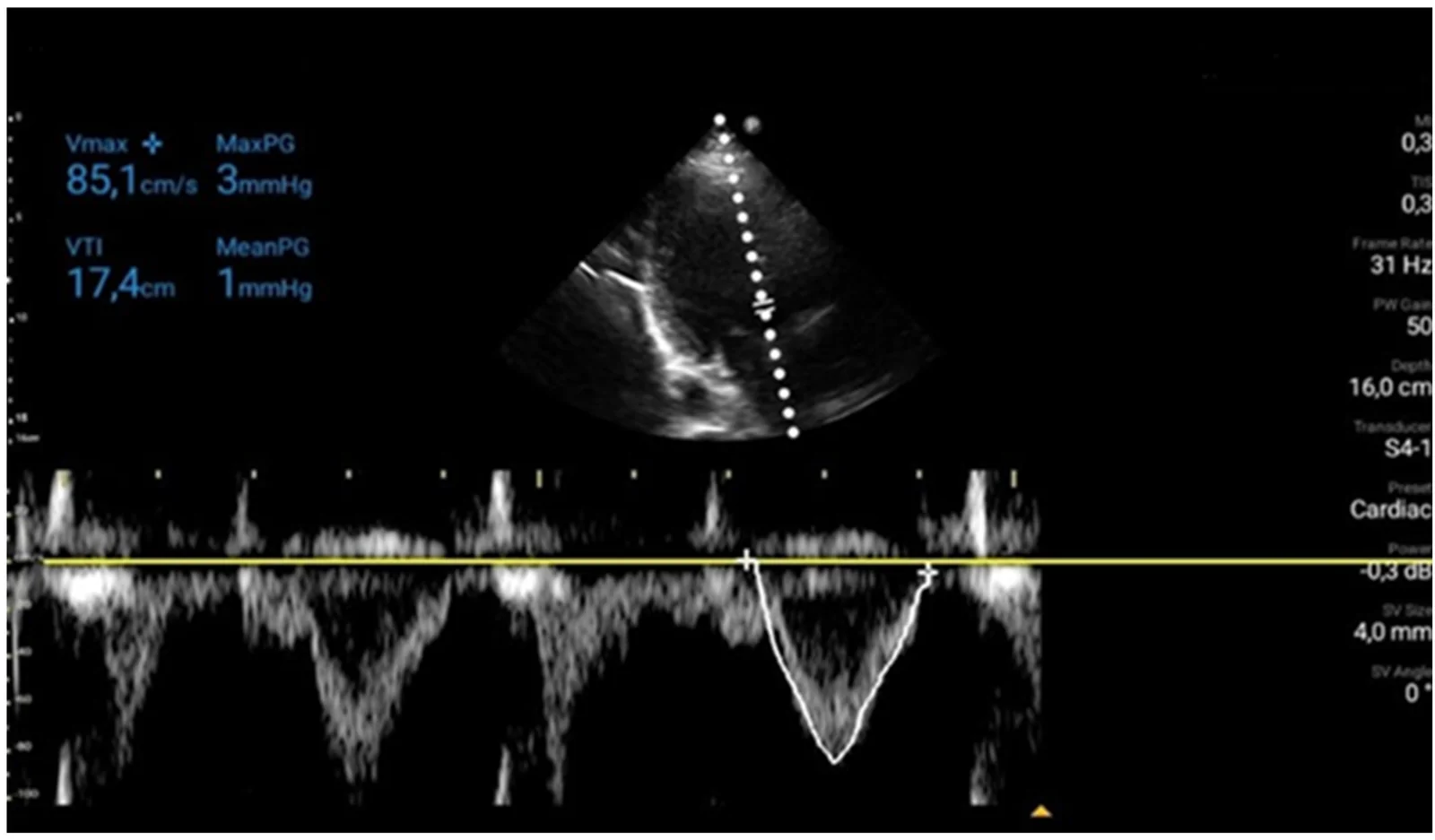
LVOT VTI measurement by pulsed-wave Doppler. Authors’ own archive. Dots line: 3–5 mm proximal of Aortic valve point, Triangle; VTI.

Echocardiographic assessments were performed by emergency physicians trained in advanced point-of-care ultrasound. The LVOT VTI was measured from the apical five-chamber view with a pulsed-wave Doppler gate placed 3–5 mm proximal to the aortic valve (Figure 2). Three consecutive cardiac cycles were averaged in sinus rhythm.

The MFC protocol was defined as follows [6]:

- Baseline VTI (VTI_1_): measured upon arrival.
- Challenge: infusion of 100 mL isotonic saline over one minute.
- Post-challenge VTI (VTI_2_): measured one minute after completion of the infusion.

The percentage change was calculated as

%ΔVTI = 100 × [(VTI_2_ − VTI_1_)/VTI_1_]

Fluid responsiveness classification; Using the meta-analytic threshold reported by Messina et al. [5], patients with ΔVTI ≥ 10% were classified as fluid responders and those with ΔVTI < 10% as non-responders. In UGPG, responders received an additional crystalloid bolus (250–500 mL) and/or blood products when indicated, whereas non-responders underwent a restrictive fluid strategy and were considered for early vasopressor support.

### 2.6. Outcomes

The primary outcome was the feasibility of ΔVTI acquisition in the ED, defined as successful completion of paired VTI_1_/VTI_2_ measurements within the protocol-specified time window in an enrolled patient. Secondary, exploratory outcomes were the association of ΔVTI with in-hospital mortality within each group, and the discriminative performance of ΔVTI for in-hospital mortality.

### 2.7. Etiology Stratification

All patients underwent upper endoscopy within 24 h of admission when clinically feasible. Etiology was classified as variceal or non-variceal; non-variceal lesions were further graded using the Forrest classification. Etiology-stratified ΔVTI and outcome analyses are presented descriptively where sample size permitted.

### 2.8. Statistical Analysis

All analyses were performed using SPSS 20.0 (IBM Inc., Chicago, IL, USA). Normality of continuous variables was assessed with the Kolmogorov–Smirnov test. Continuous variables are presented as mean ± standard deviation or median (minimum–maximum) according to distribution; categorical variables as counts and percentages. Categorical variables were compared with the chi-square or Fisher’s exact test. Normally distributed continuous variables were compared with the independent-samples *t*-test and non-normally distributed variables with the Mann–Whitney U test.

The discriminative performance of ΔVTI for in-hospital mortality was evaluated by receiver-operating-characteristic (ROC) analysis, with the area under the curve (AUC) and 95% confidence intervals reported for the whole cohort and for each group separately. The optimal cut-off was determined by the Youden index and compared with the a priori 10% threshold.

Because multiple laboratory parameters were compared by survival status within each group, a Bonferroni correction was applied to the laboratory comparisons only (pre-specified adjusted α = 0.005). Vital-sign comparisons and all other analyses were evaluated at the conventional α = 0.05 and were not subject to this correction.

A multivariable logistic regression model was constructed with in-hospital mortality as the dependent variable and ΔVTI, age, baseline haemoglobin, and bleeding etiology (variceal vs. non-variceal) as covariates.

All analyses were two-tailed, and *p* < 0.05 was considered statistically significant, except where the Bonferroni-adjusted threshold applied as specified above.

## 3. Results

### 3.1. Feasibility

Eighty-five patients with UGIB were enrolled and allocated to UGPG (*n* = 47; 55.3%) or SCPG (*n*= 38; 44.7%). Paired VTI_1_/VTI_2_ measurements were successfully obtained within the protocol-specified window in all enrolled patients, indicating that the MFC can be executed within routine ED workflow without dedicated sonographic support.

### 3.2. Demographic Characteristics and Baseline Vital Signs

Mean age was 64.5 ± 16.3 years in UGPG and 69.3 ± 13.6 years in SCPG (*p* > 0.05), and sex distribution was comparable. Systolic blood pressure (SBP), diastolic blood pressure (DBP), and mean arterial pressure (MAP) at admission did not differ between groups. Baseline oxygen saturation (SpO_2_) was statistically higher in UGPG (98.3 ± 1.5% vs. 97.0 ± 1.5%; *p* < 0.001). This imbalance is treated as a marker of underlying group non-equivalence rather than as an isolated finding, and is examined in the Discussion (Table 1).

### 3.3. Bleeding Etiology and Endoscopic Findings

Non-variceal bleeding predominated in both groups (UGPG 41/47, 87.2%; SCPG 30/38, 78.9%; *p* = 0.30). Among non-variceal cases, peptic ulcer disease with Forrest grades Ib–IIb accounted for 56/71 (78.9%). Variceal bleeding accounted for 6/47 (12.8%) in UGPG and 8/38 (21.1%) in SCPG. Sample size within strata was insufficient for inferential testing, and stratum-specific results are descriptive only (Table 1).

Table 1 Baseline demographic characteristics, admission vital signs, and bleeding etiology were compared between the two allocation groups (UGPG vs. SCPG). Continuous variables were compared with the independent-samples *t*-test and categorical variables with the chi-square test or Fisher’s exact test. Forrest-grade percentages are calculated within the non-variceal subgroup of each column (UGPG *n* = 41; SCPG *n* = 30; total *n* = 71); all other percentages are calculated within the column total. Cells marked “–” were not tested owing to insufficient cell counts. Bold indicates *p* < 0.05.

### 3.4. Predictors of Mortality Within Each Group

Associations with mortality were examined separately within each group; no comparison in Table 2 is made across groups. In SCPG, non-survivors had significantly lower admission SBP than survivors (113.0 ± 17.6 vs. 129.8 ± 23.6 mmHg; ***p* = 0.020**); as a vital-sign comparison this was not subject to the Bonferroni correction. Among the laboratory parameters, which were Bonferroni-corrected, troponin in UGPG (***p* < 0.001**) and LDH in SCPG (***p* < 0.001**) retained significance at the adjusted threshold, whereas the ALT findings in both groups (***p* = 0.04**) and the AST finding in SCPG (***p* = 0.05**) did not and should be regarded as exploratory (Table 2).

Table 2 Admission vital signs and laboratory parameters compared by survival status within each group separately (UGPG survivors vs. UGPG non-survivors; SCPG survivors vs. SCPG non-survivors). No between-group comparison is made in this table. Continuous variables were compared with the independent-samples *t*-test or Mann–Whitney U test as appropriate. Laboratory parameters only were Bonferroni-corrected for multiple testing (adjusted α = 0.005); values marked ‘*’ retained significance at this adjusted threshold. Vital-sign comparisons were evaluated at α = 0.05 and are marked ^‡^. Bold indicates statistical significance at the applicable threshold.

Hemodynamic Parameters and Fluid Responsiveness (ΔVTI)

Tachycardia (≥100 bpm) at admission was associated with mortality across the whole cohort (21.2% vs. 4.1% in normocardia; *p* = 0.030).

**Figure 3:**
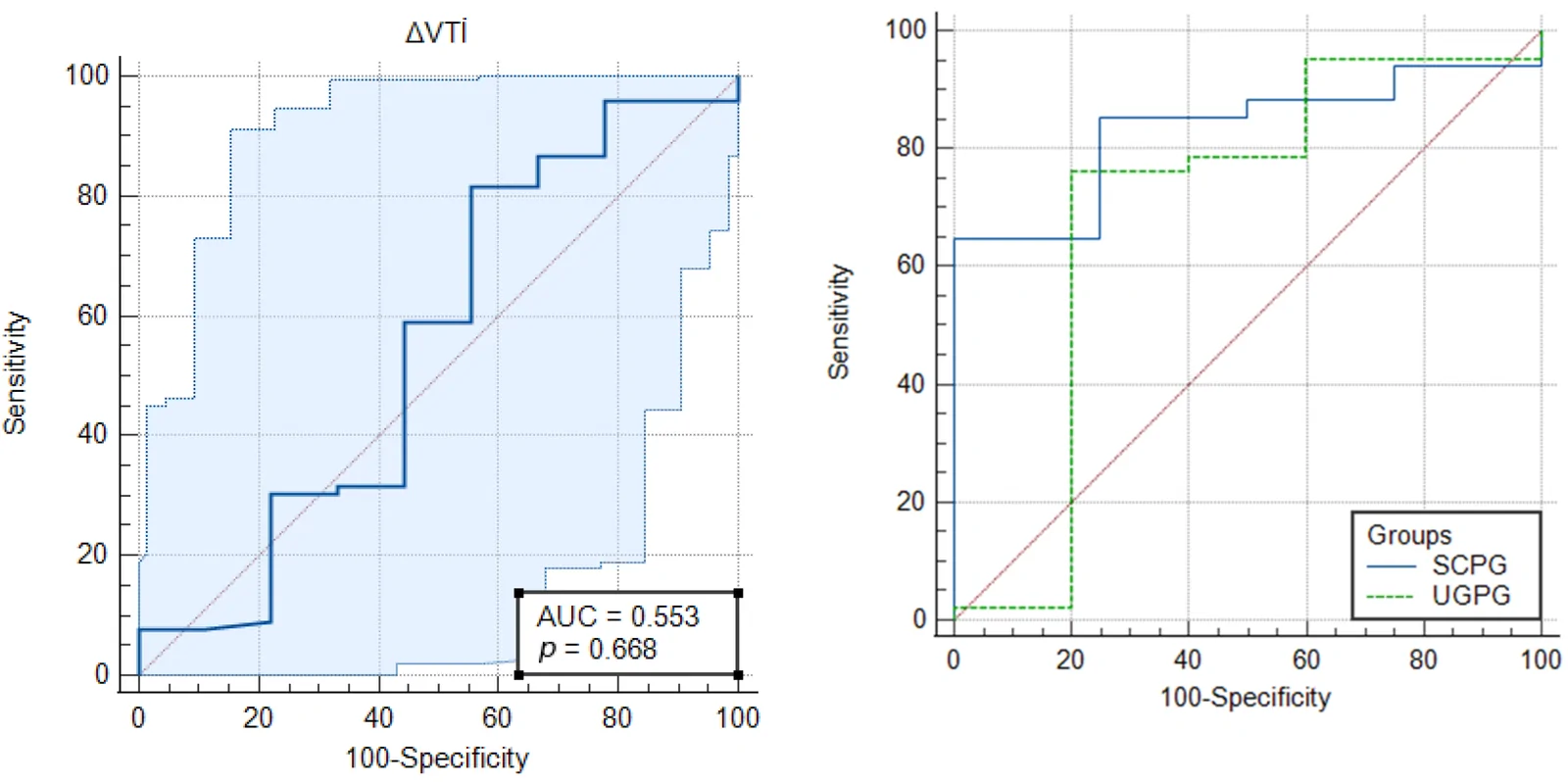
ROC curves for ΔVTI and in-hospital mortality and by groups.

ΔVTI and mortality: For the whole cohort, ΔVTI discriminated non-survivors from survivors poorly (AUC 0.55, 95% CI 0.44–0.66; the confidence interval includes 0.50, indicating no better-than-chance discrimination overall). Discrimination differed substantially by group: AUC 0.83 (95% CI 0.67–0.93) in SCPG and 0.69 (95% CI 0.54–0.82) in UGPG. The Youden-optimal cut-off in the whole cohort was 50.5% (sensitivity 44.4%, specificity 81.5%), well above the a priori 10% threshold and derived from only nine events; it should be regarded as unstable and is not proposed for clinical use (Figure 3).

In SCPG, non-survivors had significantly higher mean ΔVTI than survivors (58.6 ± 30.5% vs. 28.4 ± 30.9%; *p* = 0.030). In UGPG the categorical analysis at the 10% cut-off showed the inverse pattern, with mortality concentrated among non-responders (*p* = 0.020) (Table 3).

Heart-rate categories are compared across the whole cohort (*n* = 85); ΔVTI comparisons are made within each group separately, between survivors and non-survivors. No between-group ΔVTI comparison is made. Continuous ΔVTI values are mean ± SD; categorical ΔVTI is dichotomised at the a priori 10% threshold. Bold indicates *p* < 0.05.

*Between-group mortality comparison*: In-hospital mortality was 5/47 (10.6%) in UGPG and 4/38 (10.5%) in SCPG (*p* > 0.99). Because this study was not powered for mortality, these figures are reported for descriptive completeness only. They do not constitute a comparison of treatment strategies, no formal significance test is presented for them, and no inference regarding the superiority or non-inferiority of ΔVTI-guided resuscitation can be drawn from them.

*Multivariable logistic regression:* With ΔVTI, age, baseline haemoglobin and etiology entered simultaneously, ΔVTI was not an independent predictor of mortality. As detailed in the Statistical Analysis section, the events-per-variable ratio of approximately 2.3 places this model well below the accepted threshold for stable estimation; the coefficients are reported for transparency and must not be interpreted as effect estimates.

### 3.5. Impact of Transfusion Volume on Laboratory Markers

Patients requiring ≥2 units of erythrocyte suspension (ES) had significantly lower baseline haemoglobin in both groups (*p* < 0.001). In UGPG, higher transfusion requirement was also associated with elevated urea (*p* = 0.03) and BUN (*p* = 0.03); this association was not observed in SCPG (Table 4).

Table 4 Admission laboratory parameters compared by transfusion volume within each group separately (<2 units vs. ≥2 units of erythrocyte suspension). No between-group comparison is made in this table. Values are mean ± SD. Comparisons were made with the independent-samples *t*-test or Mann–Whitney U test as appropriate. These are exploratory sub-analyses and were not corrected for multiple testing; bold indicates nominal *p* < 0.05.

## 4. Discussion

To our knowledge, this is the first prospective evaluation of the echocardiographic mini fluid challenge in an undifferentiated ED population with acute UGIB. Two observations emerge. The technique proved executable at the bedside within routine ED workflow, which was this study’s primary question. Beyond that, ΔVTI carried prognostic information whose direction depended on whether clinicians could see it: where the measurement was blinded, death was associated with a high ΔVTI; where it was acted upon, death was associated with a low one. This inversion is the most interesting feature of the dataset and is, we argue, mechanistically coherent rather than paradoxical.

### 4.1. Why the Direction of the ΔVTI–Mortality Association Differed Between Groups

The premise of any dynamic preload test is that a positive result identifies a ventricle operating on the ascending limb of the Frank–Starling relationship, in which additional preload will be converted into additional stroke volume [5,6]. In haemorrhagic shock, this state is common early and is precisely what resuscitation is intended to abolish. The consequence is that the prognostic meaning of a positive test is entirely contingent on whether it is subsequently treated, a point that is easily lost when fluid-responsiveness indices are treated as prognostic biomarkers rather than as decision instruments.

In the standard-care group, the measurement was recorded but concealed, so a positive test triggered no change in management. Under those conditions, a high ΔVTI identifies a patient who remained preload-dependent throughout the resuscitation window: recruitable stroke volume existed and was not recruited. The mechanism linking this to death is oxygen debt. Arterial pressure is defended in early haemorrhage by baroreflex-mediated arteriolar vasoconstriction and tachycardia, so cardiac output and therefore systemic oxygen delivery can fall substantially while blood pressure remains within the range that clinicians accept as adequate [10,11,12,13]. Because VTI is a measure of stroke volume and blood pressure is a measure of the product of flow and resistance, the two dissociate exactly where clinical decision-making is most vulnerable. That the same patients showed lower admission systolic pressure only at a group level, and that heart rate identified risk with high sensitivity but poor specificity, is consistent with this: the conventional signs detect the compensatory response, not the deficit driving it.

In the ultrasound-guided group, the same test was visible and acted upon, and the interpretation changed accordingly. If responsiveness is detected and corrected, it is largely removed from the causal path to death, and the patients who die are those for whom fluid was never the answer. A ΔVTI below 10% should not be read as evidence of adequate volume status; it means only that the ventricle cannot convert further preload into output, and there are at least three distinct routes to that state. The first is genuine preload adequacy. The second is impaired ventricular compliance, in which the pressure–volume relationship is shifted such that the plateau is reached at a low absolute intravascular volume; our own data are compatible with this, in that troponin was the single laboratory variable that survived correction for multiple testing in this group, with non-survivors showing markedly higher values. Demand-ischaemic myocardial injury during haemorrhagic shock—supply–demand mismatch in the setting of anaemia, tachycardia and hypotension, that is, type 2 myocardial infarction—produces both diastolic stiffening and systolic impairment, and would render a patient non-responsive while profoundly under-resuscitated [14]. The third is a shock phenotype that is not primarily hypovolaemic: distributive shock from bacterial translocation and spontaneous bacterial peritonitis in decompensated cirrhosis, or from the systemic inflammatory response to large-volume blood in the gut, will not respond to preload augmentation and carries its own high mortality [15].

Distinguishing these three is not possible with ΔVTI alone, which is the principal conceptual limitation of a single-parameter approach and the reason we did not prospectively phenotype shock in this cohort. A patient with a low ΔVTI and a hyperdynamic, small, vigorously contracting left ventricle is a very different problem from one with a low ΔVTI and a dilated, poorly contractile ventricle, yet the two are indistinguishable on the index alone. Integrating ΔVTI with left ventricular systolic function, inferior vena cava behaviour, lung B-line assessment and lactate clearance would allow these to be separated, and would convert a binary responder/non-responder classification into an actionable phenotype [16,17].

This interpretation must be held loosely. The ultrasound-guided group contained five deaths, of which two occurred among non-responders; an association resting on two events cannot support a mechanistic claim and is presented as a hypothesis for prospective testing, not as a finding. We note it because the alternative (treating the inversion as noise) would discard the one observation in this dataset with a coherent physiological explanation.

Admission SpO_2_ differed between groups at a conventional significance threshold. The absolute difference of 1.3 percentage points is clinically negligible on its own terms: both group means lie on the flat upper portion of the oxyhaemoglobin dissociation curve, where a saturation change of this magnitude corresponds to a trivial difference in arterial oxygen content and cannot plausibly influence mortality. The statistical significance reflects small within-group variance rather than clinical separation, and illustrates why significance testing of baseline characteristics is of limited value.

Its importance is nonetheless real, but indirect: it is evidence that the two groups were not exchangeable. Marginally lower saturation in the standard-care group is compatible with a higher burden of aspiration, of chronic pulmonary disease, of hepatopulmonary shunting in cirrhosis, or simply with the higher mean age of that group. If it marks a systematically sicker population, the direction of bias would be against the standard-care group. We cannot exclude this, and it is one of several reasons why the between-group mortality figures in this study should not be interpreted as a treatment comparison. It does not, however, threaten the within-group analyses, which are the basis of every inference we draw.

Variceal and non-variceal bleeding differ not only in prognosis but in the risk–benefit calculus of volume loading. In portal hypertension, restoration of intravascular volume raises portal pressure and may reopen a bleeding varix, so the same positive fluid-responsiveness result carries a different net expected benefit than it would in a bleeding duodenal ulcer [4,9]. It follows that a single 10% threshold applied indiscriminately is a simplification, and that the clinically relevant question is not whether a patient is responsive but whether the response is worth its cost in that lesion. Our cohort was predominantly non-variceal and the variceal subgroup was too small for separate inferential analysis; this is a limitation and also a specification for future trials, which should pre-stratify by etiology rather than adjust for it post hoc.

### 4.2. Ancillary Observations

The association between transfusion requirement and elevated urea and BUN in the ultrasound-guided group is consistent with the established dual mechanism of azotaemia in UGIB and is best read as a marker of haemorrhage severity rather than as an independent finding [18]. The near-equal sex distribution in our cohort contrasts with the male predominance reported by Rațiu et al. and Aktaş et al. [19,20]; with 85 patients this difference is as likely to reflect sampling as any regional or demographic effect, and we do not attach interpretive weight to it.

### 4.3. Clinical Implications

A mini fluid challenge with Doppler VTI measurement can be performed in an ED on patients with active gastrointestinal haemorrhage, without dedicated sonographic support and within the time constraints of an initial resuscitation. It does not establish that doing so improves survival, and the present design cannot answer that question. What it does provide is a defensible rationale for the trial that would be a randomised comparison, stratified by bleeding etiology, powered for a patient-centred outcome, and using multi-parameter POCUS phenotyping rather than ΔVTI in isolation, in which the hypothesis to be tested is that unrecognised and uncorrected preload dependence is a modifiable contributor to death in UGIB.

## 5. Limitations

The limitations of this study are substantial and constrain its conclusions more than they qualify them.

The design is quasi-experimental. Allocation having the procedure performed by an emergency physician with POCUS certification might seem like a limitation; it is actually crucial for the consistency and accuracy of the results. No statistical adjustment can recover exchangeability under this design, and the observed baseline imbalance in oxygen saturation is direct evidence that the groups were not equivalent at entry. We therefore make no causal claim, and the between-group mortality figures should be disregarded as a comparison of strategies.

This study was not powered for mortality. Nine deaths occurred in 85 patients, and the within-group analyses on which our interpretation rests are based on five and four events, respectively. Estimates derived from such numbers are extremely unstable; the ROC-derived cut-off of 50.5%, in particular, is a property of these nine events rather than of the underlying population and should not be carried forward. For the same reason, the multivariable logistic regression, with approximately 2.3 events per variable, is overfitted, and we report it only to document the absence of a demonstrable independent association rather than as an estimate.

Shock was not phenotyped prospectively. Systemic vascular resistance was not estimated, lactate trajectory was not recorded systematically, and inferior vena cava assessment was not integrated into the protocol. As argued above, this leaves the interpretation of a non-responsive ΔVTI ambiguous between preload adequacy, ventricular stiffening, and non-hypovolemic shock and is the most consequential methodological gap for future work.

Patients with chronic kidney disease and with reduced ejection fraction were excluded to avoid confounding of fluid-responsiveness measurements. These are precisely the patients in whom fluid management in UGIB is most difficult, and our findings cannot be extrapolated to them. The variceal subgroup was too small for separate analysis. The study was conducted at a single tertiary centre by physicians formally credentialled in advanced POCUS, which limits generalisability to settings where such expertise is not routinely available—a constraint that bears directly on the feasibility claim itself. Finally, inter-observer variability in VTI acquisition was not formally assessed.

## 6. Conclusions

Conventional vital signs remain useful for initial risk stratification in acute UGIB but are insufficient to guide precision volume therapy, because they report the compensatory response to hypovolemia rather than the recruitable stroke volume that resuscitation is intended to restore. This study demonstrates that the echocardiographic MFC is feasible in the emergency department in this population. It further generates, but does not confirm, the hypothesis that uncorrected preload dependence contributes to mortality: where ΔVTI was concealed from clinicians, high values were associated with death, whereas where it was acted upon, mortality was concentrated among non-responders in whom fluid could not have helped. An adequately powered, randomised, etiology-stratified trial incorporating multi-parameter POCUS phenotyping is required before ΔVTI-guided resuscitation can be recommended in routine ED practice.

## Figures and Tables

**Table 1:** Baseline demographic characteristics and vital signs of the groups.

Parameters	UGPG (*n* = 47)	SCPG (*n* = 38)	Total (*n* = 85)	*p*-Value
**Age (years)**, Mean ± SD	64.5 ± 16.3	69.3 ± 13.6	66.6 ± 15.2	>0.05
**Gender**, *n* (%)				
Male	24 (51.1%)	18 (47.4%)	42 (49.4%)	0.734
Female	23 (48.9%)	20 (52.6%)	43 (50.6%)	
**Vital Signs at Admission**, Mean ± SD				
Systolic BP (mmHg)	130.0 ± 24.4	129.9 ± 23.1	130.0 ± 23.7	0.982
Diastolic BP (mmHg)	72.6 ± 14.5	67.6 ± 14.7	70.4 ± 14.7	0.126
Mean Arterial Pressure (mmHg)	91.8 ± 15.6	87.6 ± 16.1	89.9 ± 15.9	0.231
SpO2 (%)	98.3 ± 1.5	97.0 ± 1.5	97.7 ± 1.6	**<0.001**
**Heart Rate Categories**, *n* (%)				
Bradycardia (<60 bpm)	2 (4.3%)	1 (2.6%)	3 (3.5%)	-
Normocardia (60–99 bpm)	28 (59.6%)	21 (55.3%)	49 (57.6%)	-
Tachycardia (≥100 bpm)	17 (36.1%)	16 (42.1%)	33 (38.8%)	-
**Bleeding Etiology and Endoscopic Findings**, *n* (%)				
Variceal Bleeding	6 (12.7%)	8 (21.1%)	14 (16.47%)	0.30
Non-Variceal Bleeding	41 (87.2%)	30 (78.9%)	71 (83.53%)	
**Forest Classification,** *n* (%)				
Forest 3	3 (7.31%)	2 (6.66%)	5 (7.04%)	-
Forest 2c	4 (9.75%)	2 (6.66%)	6 (8.45%)	-
Forest 2b	8 (19.51%)	7 (23.33%)	15 (21.12%)	-
Forest 2a	15 (36.58%)	13 (43.33%)	28 (39.43%)	-
Forest 1b	8 (19.51%)	5 (16.66%)	13 (18.30%)	-
Forest 1a	3 (7.31%)	1 (3.33%)	4 (5.63%)	-

**Table 2:** Comparison of admission vital signs and laboratory parameters according to survival status in UGPG and SCPG.

		UGPG (*n* = 47)			SCPG (*n* = 38)	
Parameters	Survivor (*n* = 42)	Non-Survivor (*n* = 5)	*p*-Value	Survivor (*n* = 34)	Non-Survivor (*n* = 4)	*p*-Value
**Vital Signs**						
Systolic BP (mmHg)	130.6 ± 24.1	125.2 ± 26.9	**0.620**	129.8 ± 23.6	113.0 ± 17.6	**0.020** ^‡^
Diastolic BP (mmHg)	72.2 ± 14.5	76.4 ± 14.9	0.620	68.8 ± 15.2	59.5 ± 16.8	0.200
MAP (mmHg)	91.7 ± 15.4	92.6 ± 18.6	0.800	89.1 ± 15.7	77.3 ± 15.4	0.220
SpO2 (%)	98.3 ± 1.5	97.4 ± 0.5	0.060	97.1 ± 2.2	96.5 ± 3.6	0.940
**Laboratory Parameters**						
Hemoglobin (g/dL)	9.5 ± 2.6	8.0 ± 2.1	0.210	8.9 ± 2.2	8.1 ± 0.2	0.630
Urea (mg/dL)	71.0 ± 49.0	69.0 ± 24.9	0.580	63.4 ± 32.8	65.5 ± 41.9	0.870
BUN (mg/dL)	32.7 ± 23.3	32.2 ± 11.6	0.550	29.7 ± 15.3	30.6 ± 19.6	0.870
Troponin (ng/mL)	10.1 ± 9.5	24.8 ± 7.8	<0.001 *	18.1 ± 24.2	6.5 ± 0.7	0.350
INR	1.12 ± 0.16	1.18 ± 0.16	0.300	1.19 ± 0.31	1.19 ± 0.04	0.320
AST (U/L)	20.2 ± 12.2	13.2 ± 7.9	0.220	26.7 ± 17.4	15.0 ± 11.4	**0.050**
ALT (U/L)	16.6 ± 10.5	9.0 ± 3.9	**0.040**	19.6 ± 11.2	10.2 ± 7.5	**0.040**
LDH (U/L)	180.6 ± 51.6	190.2 ± 55.0	0.640	199.1 ± 86.4	113.7 ± 26.1	**<0.001**

* after Bonferroni correction.

**Table 3:** The relationship between heart rate categories, fluid responsiveness (ΔVTI), and mortality.

	Survivor	Non-Survivor	Total	Mortality Rate	*p*-Value
**Heart Rate Categories (All Patients)**	(*n* = 76)	(*n* = 9)	(*n* = 85)		
Bradikardi (<60 bpm)	3	0	3	0%	
Normocardia (60–99 bpm)	47	2	49	4.1%	0.030
Tachycardia (≥100 bpm)	26	7	33	21.2%	
**ΔVTI Values (Mean ± SD)**					
UGPG	33.3 ± 26.8	27.6 ± 39.3	47	-	0.160
SCPG	28.4 ± 30.9	58.6 ± 30.5	38	-	0.030
**ΔVTI dichotomised at 10% (<10% vs. ≥10%)**					
UGPG	3/39	2/3	47	-	0.020
SCPG	9/25	0/4	38	-	0.230

**Table 4:** Association between Erythrocyte Suspension (ES) transfusion volume and admission laboratory parameters.

	UGPG			SCPG		
Transfusion Amount	<2 Units	≥2 Units	*p*	<2 Units	≥2 Units	*p*
**Hemoglobin (g/dL)**	11.1 ± 1.8	7.3 ± 1.7	**<0.001**	10.1 ± 2.0	7.4 ± 1.2	**<0.001**
**Urea (mg/dL)**	60.6 ± 44.6	82.4 ± 47.7	**0.030**	54.9 ± 32.1	72.3 ± 32.8	0.130
**BUN (mg/dL)**	27.5 ± 21.4	38.5 ± 22.3	**0.030**	25.6 ± 15.0	33.9 ± 15.3	0.130
**Troponin (ng/mL)**	12.6 ± 8.4	12.3 ± 12.4	0.500	22.3 ± 30.3	11.3 ± 12.1	0.160
**INR**	1.08 ± 0.13	1.17 ± 0.18	0.090	1.16 ± 0.35	1.21 ± 0.23	0.100
**AST(U/L)**	21.16 ± 12.3	17.68 ± 11.6	0.160	22.53 ± 11.7	28.53 ± 21.2	0.520
**ALT (U/L)**	18.8 ± 12.6	12.3 ± 5.2	0.080	17.2 ± 11.4	20.1 ± 11.1	0.350
**LDH (U/L)**	195.0 ± 48.4	167.9 ± 51.9	0.060	163.2 ± 59.8	213.2 ± 99.9	0.160

## Data Availability

The data presented in this study is available on request from the corresponding author. The data is not publicly available due to privacy and ethical restrictions.

## Abbreviations

UGIB	Upper Gastrointestinal Bleeding
ED	Emergency Department
MFC	Mini Fluid Challenge
LVOT	Left Ventricular Outflow Tract
VTI	Velocity-Time Integral
LVEF	Left Ventricular Ejection Fraction
PW	Pulsed Wave
SBP	Systolic Blood Pressure
DBP	Diastolic Blood Pressure
MAP	Mean Arterial Pressure
SpO_2_	Oxygen Saturation
ALT	Alanine aminotransferase
AST	Aspartate Aminotransferase
LDH	Lactate Dehydrogenase
BUN	Blood Urea Nitrogen
INR	International Normalized Ratio
ES	Erythrocyte Suspension
MI	Myocardial Infarction
